# Effect of larval crowding on mating competitiveness of *Anopheles gambiae *mosquitoes

**DOI:** 10.1186/1475-2875-4-49

**Published:** 2005-09-30

**Authors:** Kija R Ng'habi, Bernadette John, Gamba Nkwengulila, Bart GJ Knols, Gerry F Killeen, Heather M Ferguson

**Affiliations:** 1 Ifakara Health Research and Development Centre (IHRDC), P. O. Box 53, Ifakara, Tanzania; 2 University of Dar es Salaam, P. O. Box 35064 Dar es Salaam, Tanzania; 3 International Atomic Energy Agency (IAEA), Agency's Laboratories Seibersdorf, Seibersdorf A-2444, Austria; 4 Laboratory of Entomology. P.O. Box 8031, 6700 EH, Wageningen University, Wageningen, The Netherlands; 5 Department of Public Health and Epidemiology, Swiss Tropical Institute, Basel, Switzerland

## Abstract

**Background:**

The success of sterile or transgenic *Anopheles *for malaria control depends on their mating competitiveness within wild populations. Current evidence suggests that transgenic mosquitoes have reduced fitness. One means of compensating for this fitness deficit would be to identify environmental conditions that increase their mating competitiveness, and incorporate them into laboratory rearing regimes.

**Methods:**

*Anopheles gambiae *larvae were allocated to three crowding treatments with the same food input per larva. Emerged males were competed against one another for access to females, and their corresponding longevity and energetic reserves measured.

**Results:**

Males from the low-crowding treatment were much more likely to acquire the first mating. They won the first female approximately 11 times more often than those from the high-crowding treatment (Odds ratio = 11.17) and four times more often than those from the medium-crowding treatment (Odds ratio = 3.51). However, there was no overall difference in the total number of matings acquired by males from different treatments (p = 0.08). The survival of males from the low crowding treatment was lower than those from other treatments. The body size and teneral reserves of adult males did not differ between crowding treatments, but larger males were more likely to acquire mates than small individuals.

**Conclusion:**

Larval crowding and body size have strong, independent effects on the mating competitiveness of adult male *An. gambiae. *Thus manipulation of larval crowding during mass rearing could provide a simple technique for boosting the competitiveness of sterile or transgenic male mosquitoes prior to release.

## Background

Mosquitoes within the *Anopheles gambiae *species complex are the most important vectors of malaria in sub-Saharan Africa [1-3]. The infective bite of these mosquitoes is in large part responsible for the more than 500 million clinical attacks of malaria reported worldwide each year, resulting in more than one million deaths [4,5]. Currently the two most widely implemented vector control strategies are indoor residual insecticide spraying and insecticide-treated bednets (ITNs), both of which have proven effective in the reduction of malaria transmission in some areas [6-11]. However, multiple insecticide resistance is emerging amongst the major malaria vectors *An. gambiae *[12] and *Anopheles funestus *[13], and there are complications associated with introduction, distribution and proper use of ITNs [14,15] that indicate these strategies alone may not be sufficient to eliminate malaria transmission. New tools aimed at stopping malaria development in humans are promising, but the development of an efficacious antigen for vaccine production is slow, and parasite resistance to locally available drugs is increasing whilst new drugs that are effective are often unaffordable [16].

One promising new control prospect is the possibility of rendering wild vector populations less susceptible to infection by releasing mosquitoes that are genetically modified to resist infection [17-19], or sterile males that will mate with wild females and stop them from reproducing [20]. In the case of a genetically modified mosquito (GMM) strategy, malaria could be reduced by fixing a resistance gene in vector populations, [21-23], and in the case of sterile male release, malaria could be cut by a collapse in the vector population due to a high frequency of unviable matings. Any such release of sterile or GM mosquitoes should consist only of males [20,24] because this sex does not blood feed, and thus they will not increase the number or nature of mosquito bites per person at release sites. The success or failure of a GMM or sterile programme will depend largely on whether released males can successfully compete for mates against wild males [25,26]. Current evidence from laboratory experiments suggests that GMMs have reduced competitiveness and are generally out-competed in the presence of unmodified laboratory-reared males [27-29]. Operationally, the consequences of releasing males with poor competitiveness are dire. For example, the general failure of mosquito control programmes launched in the 1970s that aimed to reduce vector populations by releasing sterile males can be largely attributed to their poor mating competitiveness [20,24], and to a lesser extent, the dispersal of fertile males into control areas. In the case of GMM, some argue that even if modified males have lower fitness than the wild type, refractory genes will still spread provided they are linked to an efficient genetic drive mechanism [30]. However, such a drive mechanism could only act if insemination occurs in the first place [24], which it may not if GMM competitiveness is very low. Furthermore, no efficient genetic drive mechanism has yet been identified for *Anopheles*, and even assuming one is, there are doubts about whether it could be tightly linked to a potentially costly resistance gene [23,31,32]. The enhancement of male competitiveness thus remains crucial for successful gene introduction. Gaining an understanding of the ecological factors that govern *Anopheles *mating biology in general, and promote male competitiveness in particular, will increase the chances of success of future GMM and sterile male-based control efforts [24-26,33].

One ecological factor known to have a great influence on the life-history of adult Anopheline, Culicines and Aedes mosquitoes is the density at which larvae develop [34-37]. In nature, larvae of *An. gambiae *hatch and grow in a range of aquatic habitats [38]. In the absence of predators and pathogens, the number of larvae in a particular habitat and the amount of food available to them determines the number of adults that emerge from a habitat [39,40], their survival [37,40] and body size [34,41,42]. Crowded larvae are thought to be at a disadvantage because they are faced with greater competition for food [40], and are exposed to higher levels of toxic waste products, crowding chemicals and physical interference from other larvae [43-45].

Whereas the importance of larval density to female *Anopheline *and *Aedes *mosquitoes has been broadly investigated [34,46,47], no doubt prioritized because of their direct role in disease transmission, little is known about its consequences for male mosquito vigour. Of the few known studies (in *Anopheles *and *Aedes sp*.) that have considered how larval density could influence male development [41,48,49] their focus has been on the effect of food limitation, not that of chemical or physical interference.

Here the effect of larval crowding on the mating competitiveness of adult male *An. gambiae *was investigated. The focus was specifically on the effects of crowding in larval habitats, not on food limitation, which was controlled for by providing each larva with an equal amount of food per unit time. Crowding was prioritized for study because, space rather than food was believed to be the biggest limiting factor when mass-producing transgenic or sterile mosquitoes for field release. In addition to conducting mating assays, the teneral reserves of males from different crowding conditions was also quantified to test if any observed differences in competitiveness could be explained by energetic limitation. Energy reserves influence mosquito behavioural activities such as swarming and feeding [50,51], and may vary in response to larval crowding. In addition to testing the effect of larval crowding on mating competitiveness, it was also examined whether it influences male longevity, as this is another potential determinant of male lifetime reproductive fitness.

## Methods

### Rearing

*An. gambiae sensu stricto *from a colony at the Ifakara Health Research and Development (IHRDC), Tanzania, were used in this study. This colony was established from a wild population located near Njage village in 1996. First instar *An. gambiae s.s. *larvae were obtained from colony cages and assigned randomly to density treatments of 100, 200 and 300 larvae per rearing tray (37 × 14 × 13 cm). Each tray was filled with 1 L of water and supplied with fish food (Tetramin^®^). In each tray, 0.2 mg of Tetramin^® ^was added for each larva, thus 20 mg, 40 mg or 60 mg was added to the low-, medium- and high-crowding treatment trays respectively, each day. Trays were inspected visually twice a day for the presence of pupae. Once detected, pupae were collected, counted and held individually in vials to allow for emergence. Batches of males from all three larval treatments that emerged on the same day were compared against one another in mating trials using females from the low-crowding treatment.

### Marking

From the time of emergence, males were pooled according to crowding treatment and held in separate cages. On the second day after emergence, cohorts of adult males from two of the three rearing conditions were marked with green or pink fluorescent dusts respectively. One group was left unmarked. Marking treatments were alternated between crowding treatments across trials to ensure no systematic bias in performance due to dusting. Furthermore, pilot studies where males from the same crowding condition were marked with different colours revealed no effect of dust presence or colour on mating performance.

### Mating experiments

On the third day after emergence, 30 males (10 males from each crowding treatment) were put together in one cage (15 × 15 × 10 cm). The cage was exposed to natural light a few hours before dusk. Observation of the cage began approximately 10 minutes prior to dusk. One or two males were observed to initiate the swarming process, just above a black disc (a swarm marker) that was placed on the bottom of the cage, with most of the remaining males joining the swarm after a few minutes. Once swarming was underway, 10 females from the low crowding condition were added to the cage (making a 3:1 male to female ratio). These females were simultaneously released into the cage using an aspirator. Mating activity was observed with a low-watt red light bulb. Pairs observed to form copula were immediately aspirated out of the cage and put together into a holding cup. On each evening of experiments, observation of mating was confined to an interval of 40–45 minutes. Observation ceased when all males had stopped swarming. At the close of the swarming session, unmated females were removed from the cage. The following morning, a fluorescent lamp was used to identify the larval rearing environment of each mated male.

Females observed to have copulated with males were blood-fed on the morning following mating and moved into individual vials. Wet filter paper was placed on the bottom of these vials to act as an oviposition site. After five days in individual holding tubes, all eggs laid by mated females were collected and counted. Wing lengths of both males and females that mated, as well as a sub-sample of those from males that did not, were measured under a dissecting microscope.

### Quantification of energy reserves

Batches of newly emerged males from each larval crowding regime were killed by shaking and transferred individually into glass test tubes for the quantification of lipids, sugars and glycogen. Once in tubes, mosquitoes were crushed using a glass rod. One hundred micro-litres (μl) of 2% sodium sulphate (which adsorbs glycogen) and 600 μl of a 1:2 chloroform-methanol mixture (which dissolves lipids and sugars respectively) were added to each tube. Tubes were then covered and incubated for 24 hrs at room temperature. For each batch of males that was analysed, one blank was prepared by adding the same chemicals to a tube that had no mosquito. Lipids, glycogen and sugars of each male, were then quantified using a colorimetric technique adapted for mosquito analysis [52].

### Longevity of unmated males

In a separate series of experiments, males emerging from each larval rearing regime were denied access to females but held in cages to monitor their longevity. These males were provided with a 10% glucose solution for sustenance until death. All dead males were removed and counted daily.

### Statistical analyses

The main aim of statistical analyses was to test for differences in the mating competitiveness, energy reserves, and longevity of *An. gambiae *males reared under different crowding conditions. Three analyses were conducted to assess mating competitiveness. First, analysis was restricted only to the first male to mate in each of 28 trials. The first male to mate was considered to be the fittest in the group (the first place 'winner'), and used a chi-square test to examine how larval crowding treatment influenced a male's probability of being a winner. Secondly, to test whether the total number of copulations in all nights was influenced by larval crowding treatment, a chi-square test was again used. Finally, the order in which males mated during a night (1^st^, 2^nd^, 3rd etc.) was examined whether was influenced by larval crowding treatment. For this, the analysis was restricted to data from the 14 trials (out of 28) where at least five matings occurred in a night. Males that mated were given a rank that corresponded to the order in which they mated during the trial (e.g. 1^st ^to mate got '1', etc). A Kruskal-Wallis test was then used to test the relationship between larval crowding treatment and mating rank (dependent variable). General Linear Models (GLM) were used to test whether larval crowding treatment influenced male wing length, or the abundance of lipids, glycogen and sugars they had on emergence. GLM were also used to test whether the number of eggs laid by a female was influenced by the larval crowding condition of the male that inseminated her. Finally, Kaplan-Maier survival analysis was used to test whether the survival of males depended on the crowding condition under which they were reared. All statistical analyses were done using the SPSS for windows and SAS system for Windows (version 8).

## Results

### Mating competitiveness

**Figure 1:**
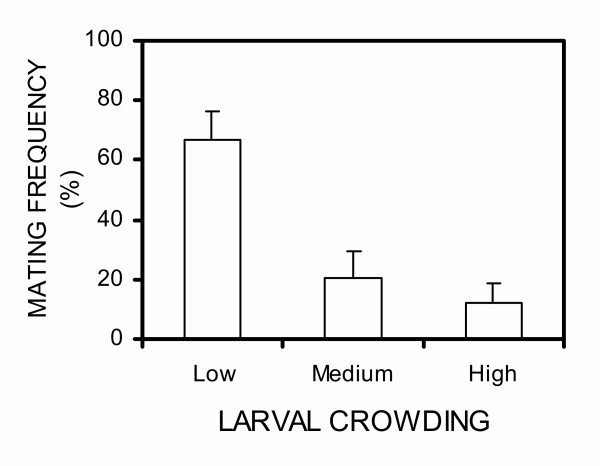
Frequency at which males from high, medium and low crowding conditions were the 'first-to-mate' in 28 nights of mating trials. The error bars represent the standard error as estimated from the binomial distribution.

**Figure 2:**
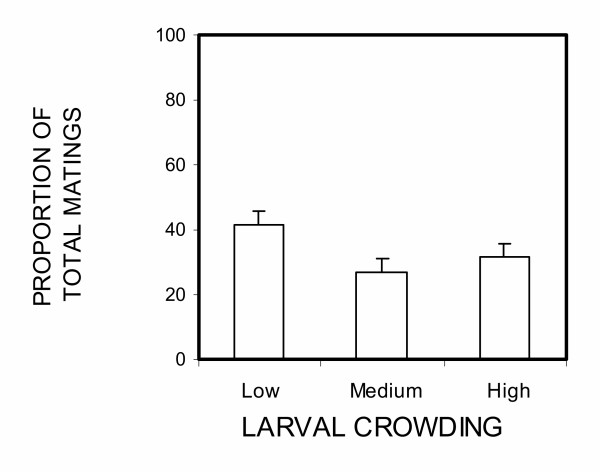
Proportion of total matings in 28 nights of trials going to males from low, medium and high larval crowding treatments. Error bars are the standard error as estimated from the binomial distribution (n = 133).

**Figure 3:**
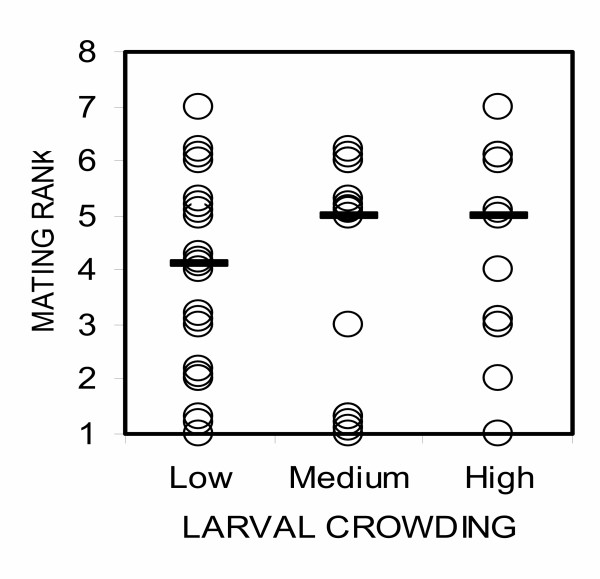
Distribution of mating ranks of males from low, medium and high crowding treatments as observed in the 14 mating trials in which at least 5 matings occurred. One circle represents two observations and the dark line in each treatment gives the median mating rank. Overlapping lines have the same mating rank, but have been spaced to indicate the number of observations per rank.

A total of 1,120 *An. gambiae *mosquitoes were used in 28 nights of mating experiments (280 females and 840 males). Restricting consideration to the first male to mate, we observed that males from low crowding environments were much more likely to succeed (χ_2_^2 ^= 13.61, p = 0.01, Figure. 1). Males from the low crowding treatment won approximatelly11 times (Odds ratio [95% CI] = 11.17, [2.7–50]) more often than those from the high crowding treatment, while those from the medium crowding condition won approximately 4 times more often (O.R [95% CI] = 3.51, [0.9–16.7]). Analysis of all copulations (not just the first) in all 28 nights trials showed no statistically significant difference in mating frequency between males from different crowding treatments (χ_2_^2 ^= 4.99, p = 0.08), however there was a trend towards a higher mating frequency at low crowding condition, similar to that demonstrated in the 'first-to-mate' analysis (Figure 2). In the subset of 14 trials where at least five males mated, there was a weak tendency for males from the least crowded larval condition to mate before those from more crowded conditions, but it was not statistically significant (χ^2^_2 _= 5.09, p = 0.08, Figure. 3).

**Figure 4:**
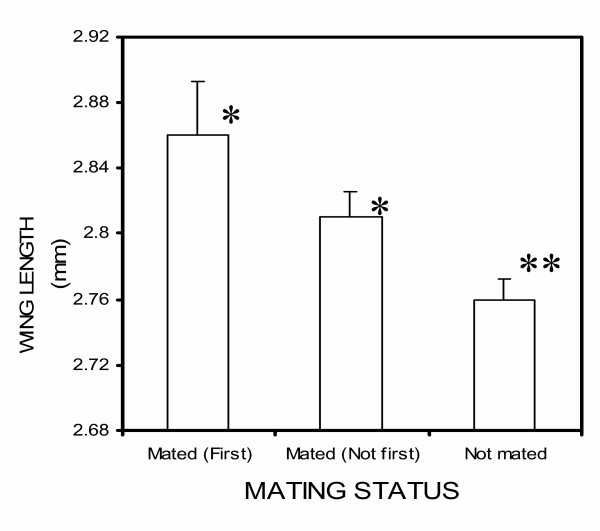
Body sizes (as indexed by wing length) of males who were the first to mate, who mated but were not the first, and that did not mate at all. Bars with the same number of asterisks (*) are not statistically different, but bars with differing numbers are.

The average size of male mosquitoes did not vary significantly between larval crowding treatments (F_2,397 _= 2.43, p = 0.09, mean body size: 2.75 ± 0.23 mm, 2.79 ± 0.12 mm and 2.79 ± 0.12 mm for low-, medium- and higher-crowding conditions, respectively). However, of those that were measured (n = 398), males who successfully obtained a female were larger than those that did not (F_1,397 _= 6.97, p = 0.01, mean body size: 2.82 ± 0.02 mm and 2.76 ± 0.01 mm respectively, Figure. 4). There was no difference between the body size of males who mated first, and those who mated later in the evening (F_1,115 _= 1.79, p = 0.18, but both groups were larger than males who did not mate, Figure. 4).

Only 15 out of 52 mated and subsequently blood-fed females oviposited their eggs. Amongst this subset, no association was found between egg batch size and paternal crowding condition (F_2,12 _= 0.67, p = 0.53) or maternal wing length (F_1,11 _= 1.98 p = 0.19). Additionally, there was no association between the probability that females would oviposit and the larval crowding condition of her mate (χ_2_^2 ^= 0.91, p = 0.63).

### Male teneral reserves and longevity

**Figure 5:**
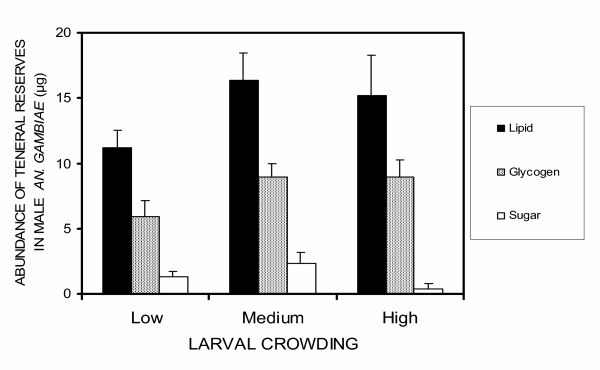
The mean mass of lipids, glycogen and sugar in newly emerged *An. gambiae s. s. *males reared in low, medium and high larval crowding conditions.

Pooling all treatments, the mean amounts of teneral reserves in newly emerged males were 14.24 (± 1.34) μg of lipids, 1.34 (± 0.71) μg of sugars and 7.96 (± 0.39) μg of glycogen. There was no evidence that larval crowding conditions influenced the abundance of these reserves in newly emerged adult males (lipids: F_2,66 _= 1.36, p = 0.26, sugars: F_2,66 _= 2.16, p = 0.12 and glycogen: F_2,66 _= 2.12, p = 0.13, Figure. 5).

**Figure 6:**
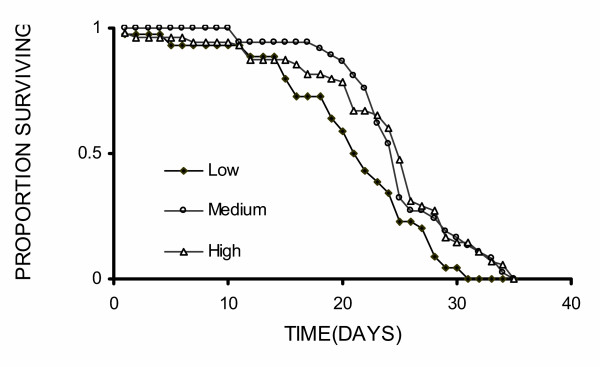
Survival of adult male *An. gambiae s.s.*, from low, medium and high larval crowding treatments.

The survival of 132 male *An. gambiae *was observed (N_low _= 44, N_medium _= 37 and N_high _= 51). The survival of adult males varied in response to the crowding conditions under which they were reared (Log-rank = 10.79, df = 2, p < 0.01, Figure 6), with the median survival of males equaling 21, 25 and 26 days for low-, medium- and high-crowding conditions, respectively. Males from low larval crowding conditions had poorer survival than those from medium (Log-rank = 7.14, df = 1, p < 0.01) and higher crowding treatment (Log-rank 8.14, df = 1, p < 0.01). The survival of males from medium and higher crowding conditions did not differ (Log-rank = 0.12, df = 1, p = 0.73).

## Discussion

This study shows that larval crowding influences the mating competitiveness of male *An. gambiae *mosquitoes. Results from 28 replicated experiments indicates that males reared under low crowding conditions are eleven times more likely to be the first in a swarming group to obtain a female than those reared at high crowding conditions. However, when all matings were considered (not just the first in each night), there was no evidence that the frequency of copulations obtained by males varied in response to larval crowding conditions.

Thus, this study has shown that larval crowding conditions influences a male's chance of beating his competitors in order to obtain the first female, but not his chance of getting a female in general. What does this say about the role of larval crowding as a determinant of male fitness? It was proposed, that a male mosquito's ability to obtain the first available female is more likely to reflect their lifetime reproductive potential than their success in eventually getting a mate; especially under controlled laboratory conditions. There are several reasons for this hypothesis. The first is that during mating, male *An. gambiae *implant a mating plug in females which presents a temporary physical barrier to further insemination [53-56]. Presuming females do not leave the swarm as soon as they are mated, males who hesitate may find themselves at a greater risk of encountering unreceptive females than those who mated first. Secondly, mating in *An. gambiae *is thought to be confined to a 15–20 min period [57-59] around dusk. Within this period, some males have been observed to return to the swarm after they have mated and continue seeking females [57]. Those who obtain the first females that enter the swarm are more likely to have sufficient time to return to the swarm after mating to look for additional females than those who mate later in the night. In our study, males were removed from the mating arena as soon as they obtained mate, so we could not test whether earlier maters were also more likely to mate repeatedly during the evening or not. However, this possibility is worth further study. A third reason for believing that those males who mated first have the highest mating competitiveness is that in nature, males are exposed to predation risks from insect predators, such as dragonflies, while swarming [60,61]. Those who mate first can leave the swarm and escape this risk, or even if they remain in the swarm, will have had the advantage of passing on their genes before being preyed upon. If predators we could have been introduced into the laboratory experimental cages, the ones that mated first might have exhibited an additional survival advantage. The final reason for hypothesizing that males who were the first to mate in our experiments would be the most competitive in nature is that the conditions under which male *Anopheles *compete for females in the field are much more intense than those created here. For example, while here the ratio created experimentally was 3 males to each female, in the field, males outnumber females at the mating site, in the range of 10:1 up to 600:1 [62,63]. Under such skewed conditions where males dramatically outnumber females, it is extremely important for a male to seize a female at the earliest opportunity, as there is no guarantee another female will turn up before the end of the evening. Thus, any factor that was believed to increases a males chance of being the 'first-to-mate', as the study demonstrated with larval crowding, will be strongly correlated with their lifetime reproductive successes under natural conditions.

Several possible mechanisms that could explain the differences in mating patterns between crowding treatments were evaluated. The first was body size, which influenced the total number of males that mated, with larger males being more likely to obtain a mate than smaller ones. A similar finding was reported in *Anopheles freeborni *[57], whereas no size-dependency for mating was observed in *An. gambiae *by Charlwood [66]. Although body size influenced mating, in general it did not explain treatment-associated differences in males who were the first-to-mate. This is because there was no difference in body size between males who mated first or later and no systematic difference in body size between crowding treatments. To conclude, both body size and larval crowding can independently influences male mating success, and that the effect of the latter is not exclusively driven by variation in the former trait.

The amount of teneral reserves in males did not differ between crowding treatments, and thus could not explain this differences in mating success. Eliminating these possibilities, it was hypothesized that the observed differences in mating success between crowding conditions could be due to the detrimental effects of chemicals [43,45] and/or waste products that are released in crowded conditions, with larvae grown in dense conditions suffering more from exposure than those at low crowding.

When held at high density, some mosquito larvae release 'crowding chemicals' that retard the growth of their conspecifics [43]. This phenomenon has been recorded for *Aedes aegypti*, but not for *Anopheles*. In *Aedes*, chemical growth retardants are released by larvae when their density increases, even if each larva receives a constant ration of food [45]. There is, however, a certain food ration threshold above which no chemicals are produced regardless of the number of individuals [44,45]. When food rations are below this threshold, however, the release of these chemicals may regulate the number of adults that emerge [43]. Although the presence of such chemicals was not assayed here, its existence would explain why in the absence of food limitation, mosquitoes grown in highly crowded conditions performed poorer than those from low crowding. The mechanism through which such chemical factors could have influenced mating success is not clear, as it was not associated with between-treatment variation in body size or teneral reserves. Thus, it was assumed that exposure to these chemical factors may have led to subtle differences in size, behaviour or physiology not detected here (i.e. changes in male flight ability or reaction time) that ultimately influenced mating competitiveness. Further experiments are required to confirm whether such chemical factors exist in *Anopheles*, and how they operate.

Larval crowding also influenced the survival of *An. gambiae *adult males (Figure 4). Whereas males from low crowding conditions were generally the first to mate and, thus, probably the most competitive for mates, they also had the poorest survival. This observation suggests the existence of an energetic trade-off between reproduction and survival in male *Anopheles*, such as has been observed in other insects [64]. In male *An. gambiae*, such a trade-off could arise because males that are the first to mate are those that are the most active, and spend more time flying and swarming than those with lower mating success. As flying is energetically costly [50,53], an increased tendency to do so may lead to both; an enhanced mating competitiveness and reduced long-term survival, as we observed in males from the low crowding condition here. As the proportion of time that males from different crowding conditions were flying in this experiment was not observed, it remains unknown if differential activity could explain the between-group variation in mating success and survival. Further study is required to measure whether flight activity is linked to mating success, and it whether influences the rate at which a male's energy reserves and longevity decrease.

The reduced survival of males from low crowding conditions may not necessarily compromise their long-term reproductive fitness. The benefits of being the first to mate during the early part of their adult life, as discussed above, may compensate for having a reduced number of mating opportunities in the longer term due to poorer survival. If so, our findings are consistent with the theoretical claim that longevity may not be a reliable measure of male reproductive fitness [64,65]. Further experiments in which males are given multiple opportunities to mate during their natural life are required to confirm whether being the first to mate on any given evening is indeed the best predictor of male mosquito lifetime reproductive success. Ideally these experiments would be carried in larger semi-field systems [33], as well as in natural populations, so realistic costs of activity (i.e. exposure to predation, energetic drain) can be incorporated.

## Conclusion

These novel findings have direct application to genetic control strategies for malaria that seek to reduce transmission by releasing sterile or malaria-refractory *Anopheles *males. The reported poor competitive success of transgenic male mosquitoes [27-29] could be enhanced by rearing males in conditions of low crowding and high food abundance. This could create a cohort of highly competitive yet relatively short-lived males for release. Ideally, transgenic males should be both highly competitive and long-lived. However, should an energetic trade-off exist between their competitiveness and longevity as suggested here, we argue it would be more useful to focus on increasing their short-term mating competitiveness by methods such as those discussed here.

To increase the competitiveness of mass-reared males, it is advocated: 1) to maintain males at low densities and/or regular changing of rearing water to avoid the build-up of crowding of chemicals that might result in disadvantaged males, and 2) to supply larvae with sufficient amounts of food. This finding, therefore, may help to overcome some of the mating-related hurdles that impeded early genetic control trials [24]. This proposes that, the fitness of all current genetically modified *Anopheles *constructs [17,19] be re-assayed after under ideal larval conditions in order to show how substantially ecological manipulation could increase their mating success relative to the wild type.

## Authors' contributions

KN and BJ were directly involved in the experimental work. KN, HF and GK developed the experimental design. HF helped in logistics, advised statistical analysis of the data and supervised manuscript preparation. BGJK obtained funding for this work. GK, BGJK and GN provided comments on the manuscript prior to submission.
